# Polyurethane-Based Coatings with Promising Antibacterial Properties

**DOI:** 10.3390/ma13194296

**Published:** 2020-09-25

**Authors:** Maurizio Villani, Federico Bertoglio, Elisa Restivo, Giovanna Bruni, Stefano Iervese, Carla Renata Arciola, Francesco Carulli, Salvatore Iannace, Fabio Bertini, Livia Visai

**Affiliations:** 1 Istituto di Scienze e Tecnologie Chimiche “Giulio Natta”—CNR, Via A. Corti 12, 20133 Milano, Italy; francesco.carulli@unimib.it (F.C.); salvatore.iannace@cnr.it (S.I.); fabio.bertini@scitec.cnr.it (F.B.); 2 Department of Molecular Medicine (DMM), Center for Health Technologies (CHT), UdR INSTM, University of Pavia, Viale Taramelli 3/B, 27100 Pavia, Italy; federico.bertoglio01@ateneopv.it (F.B.); elisa.restivo01@ateneopv.it (E.R.); stefano.iervese01@ateneopv.it (S.I.); 3 Center for Colloid and Surfaces Science (C.S.G.I.), Department of Chemistry, Physical Chemistry Section, University of Pavia, viale Taramelli 16, 27100 Pavia, Italy; giovanna.bruni@unipv.it; 4 Department of Experimental, Diagnostic and Specialty Medicine (DIMES), University of Bologna, Via S. Giacomo, 14, 40126 Bologna, Italy; carlarenata.arciola@unibo.it; 5 Laboratorio di Patologia delle Infezioni Associate all’Impianto, IRCCS Istituto Ortopedico Rizzoli, Via di Barbiano 1/10, 40136 Bologna, Italy; 6 Department of Occupational Medicine, Toxicology and Environmental Risks, Istituti Clinici Scientifici Maugeri S.p.A Società Benefit, IRCCS, Via S. Boezio, 28, 27100 Pavia, Italy

**Keywords:** silver nanoparticles, thermoplastic polyurethane (TPU), waterborne polyurethane (WPU), coatings, bactericidal surfaces

## Abstract

In coatings technology, the possibility of introducing specific characteristics at the surface level allows for the manufacture of medical devices with efficient and prolonged antibacterial properties. This efficiency is often achieved by the use of a small amount of antibacterial molecules, which can fulfil their duty while limiting eventual releasing problems. The object of this work was the preparation and characterization of silver, titanium dioxide and chitosan polyurethane-based coatings. Coatings with the three antibacterials were prepared using different deposition techniques, using a brush or a bar coater automatic film applicator, and compared to solvent casted films prepared with the same components. For silver containing materials, an innovative strategy contemplating the use and preparation of silver nanoparticles in a single step-method was employed. This preparation was obtained starting from a silver precursor and using a single compound as the reducing agent and stabilizer. Ultraviolet-visible spectroscopy, scanning electron microscopy, energy dispersive X-ray spectroscopy, contact angle measurements and adhesion test experiments were used to characterize the prepared coatings. Promising antibacterial properties, measured via direct and indirect methods, were registered for all the silver-based materials.

## 1. Introduction

Device-associated infections are one of the most common complications in medical practice. Although the development of smaller and continuous-flow devices has improved survival and reduced infection rates, one of the major causes of mortality and morbidity in these patients remains infection [1]. The treatment of medical device-related infections is challenging. The main reason is that microorganisms can adhere to the surfaces of devices and may form biofilms, i.e., aggregations between microorganisms, leading to differentiated growth, structural change and reduced protection from antimicrobial agents and different host immune mechanisms, when compared to planktonic microorganisms [2]. Once the biofilm develops it is very difficult to eradicate and the considerable cost of treatments can be due to the frequent need for prolonged hospitalization, surgery and long-term antibacterial therapy. It is reported that at least 1.7 million of annually nosocomial infections in United States are associated with biofilms and the main bacteria causing these infections worldwide are of the *Staphylococcus* species and multidrug-resistant Gram-negative bacteria such as *Escherichia coli, Klebsiella pneumoniae*, *Acinetobacter baumannii* and *Pseudomonas aeruginosa*, which is even responsible for catheter-associated urinary tract infections [2]. However, bacterial adhesion can be affected by surface properties [3,4].

Polymers are now widely used for medical device manufacturing: their easy processability, excellent mechanical properties and chemical stability make them the best candidate to be used in several biomedical applications, including catheters, different types of probe, breast implants, heart valves and vascular prostheses [5,6,7]. Polymers have a wide range of properties and the possibility to adapt their surface characteristics to different biological environments has attracted the attention of biomedical device developers. By applying specific surface modification techniques (employing biological, chemical or physical methods), it is possible to properly tailor morphological or chemical surface properties [8,9,10]. It must be noted, however, that some of these procedures, such as chemical modification, etching or roughening, could lead to weakened mechanical properties of the bulk material and/or loss of specific surface functionality.

In this respect, coating technology offers an interesting alternative to manufacturing hygienic surfaces which are capable of limiting bacterial contamination [11]. The deposition of a thin polymeric top layer starting from an opportune solution/dispersion of antibacterial components in a specific liquid system, is both easy to achieve, from a manufacturing point of view, and ensures that the coating is efficiently charged. This efficiency derives from the possibility to concentrating the antibacterial charge on the surface. Compared to other procedures used to incorporate antibacterial agents (i.e., melt compounding [12,13,14,15,16], where antibacterial agents are trapped between layers of the material constituting the device and are not all exposed to the surface) better antibacterial properties can be achieved. This maximized surface/charge ratio is obtained by starting from a lower amount of charged species and by keeping the processing simple, versatile and low-cost. Moreover, an extended life-time of the devices can be achieved due to the polymeric component of the coating which acts as a rate-limiting barrier and heightens performance in different environments by fabricating coatings with multiple antibacterial functions [17]. However, a key aspect to consider in the formulation is the deposition technique. Spray and dip coating depositions are the main techniques employed for such devices and therefore the dispersion should possess a series of characteristics which would ensure the physical and chemical properties required for their deposition and solidification over the selected substrate [18,19]. The formulation of the coating ultimately depends on the chemical compatibility between the polymer matrix and the antibacterial agent, in view of the desired activity and to prevent release contamination. The type of antibacterial activity can be selected choosing from one of these three antibacterial families: antibacterial agent release, contact killing and anti-adhesion/bacteria-repelling [20,21,22,23].

Preparation of polymeric coatings with chemically anchored contact-killing biocides or the use of a low amount of inorganic compound results in less compatibility problems. For this reason, silver nanoparticles (AgNP) represent an interesting way to prevent the rapid and excessive release of ions by ensuring a high level of antibacterial efficiency [24]. Although pure metal particles, including silver, have been extensively used for different bacterial strains, a number of disadvantages have been observed. From a processing point of view, metal particles tend to aggregate easily when mixed in a polymer matrix making their production feasible within a specific range of filler amount which, as a consequence, can have a significant impact on antibacterial activity.

Alternatively, coatings dispersions can be formulated to modify the surface energy and promote an antibiofouling effect. For example, an increased hydrophobicity results in a “slippery” surface which is capable of minimizing bacterial adhesion and biofilm formation of several bacterial strains like *P. aeruginosa*, *S. aureus*, and *E. coli* [25].

Because each antibacterial species/matrix system has unique properties, careful examination of the specific adhesion requirements for targeted application is needed. The performance and lifetimes of the coatings are strongly dependent not only on the intrinsic properties of the materials, but also on adhesion between the coating and substrate. Chemical and physical intermolecular interactions between the substrate and the top coatings help to prevent adhesion failure and ensure long-term durability [26,27].

To address these issues and to develop innovative coatings with specific antibacterial functions, we selected silver, chitosan and titanium dioxide. These antibacterials have already used as fillers for thermoplastic polyurethane (TPU)-based composites by us in a previous work [12]. Here, we use them to prepare TPU-based dispersions/solutions through a simple and versatile preparation method to produce efficient antibacterial coatings. This allows for a direct comparison between alternative preparation techniques (i.e., melt processing and post-processing).

## 2. Materials and Methods

### 2.1. Materials

The Estane^®^ 58887NAT 036, a thermoplastic polyurethane (TPU) of biomedical grade characterized by an excellent resistance to hydrolysis with excellent performance at low temperature and clarity, was supplied by Lubrizol (Wickliffe, OH, USA). Silver Nitrate (AgNO_3_), titanium dioxide anatase nanopowder (TiO_2_) with a size distribution ≤25 nm and low molecular weight chitosan (CHIT) with an average molecular weight of 50–190 kg/mol (based on viscosity) and a degree of deacetylation of 75–85%, were purchased by Sigma-Aldrich, Milano, Italy. Waterborne polyurethane (WPU) dispersed in water, with a weight-average molecular weight of 9200 g/mol and polydispersity of 17.3 was used.

### 2.2. Preparation of the Mother Liquors

Silver nanoparticle (AgNP) dispersions were prepared following these steps: 10 mL of tetrahydrofuran (THF, Sigma-Aldrich, Milano, Italy) was employed to dissolve 600 mg of TPU in order to have a TPU solution (60 mg/mL) while 2 solutions of aqueous AgNO_3_ were prepared with concentrations of 5 and 25 mM, respectively. Typically, 1 mL of each aqueous AgNO_3_ solution was added to a TPU solution and left to react under sunlight for 30–60 min in order to promote AgNP formation. TiO_2_ dispersions were similarly prepared by dispersing 3 mg of this material in TPU/THF solutions (60 mg/mL). Chitosan solutions with a concentration of 10 mg/mL were prepared by dissolving the compound in an aqueous solution (1% v/v) of acetic acid (Sigma-Aldrich, Milano, Italy).

In order to avoid the use of THF, necessary for the dissolution of the TPU, and to develop a more ecofriendly system, some experiments were performed starting with waterborne polyurethane (dispersed in water). The addition of 1 mL of AgNO_3_ aqueous solution 25 mM to 10 mL of WPU was performed.

**Figure 1:**
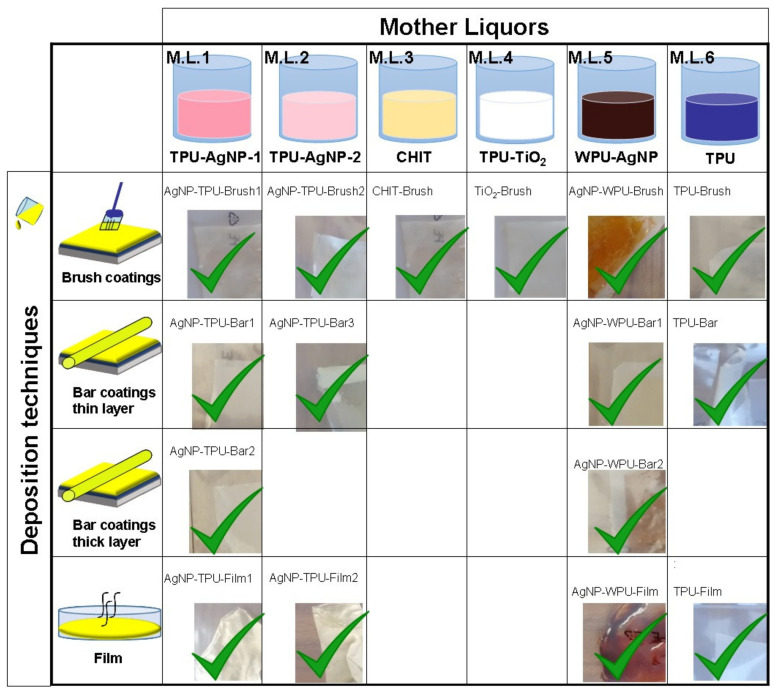
Mother liquors and relative films and coatings prepared according to the deposition techniques employed. Pictures of each sample are reported with the relative sample name.

In this way, it was possible to prepare the following antibacterial mother liquors (M.L.) (Figure 1): M.L.1 = TPU-AgNP-1 0.04 wt %; M.L.2 = TPU-AgNP-2 0.008 wt %; M.L.3 = CHIT 1 wt %; M.L.4 = TPU-TiO_2_ 0.03 wt %; M.L.5 = WPU-AgNP (all the weight percentages refer to the antibacterial content). A TPU–THF solution was treated under identical conditions for reference purposes (M.L.6). A schematic representation of the general procedure used for the preparation of the M.L. is shown in Figure S1 of the Supporting Information part.

### 2.3. Preparation of the Coatings

TPU films with fixed dimensions (5 × 5 cm^2^) were prepared by compression molding following the procedure presented in our previous work [12] and used as substrates for the coating employed.

Coatings from M.L.1 to 6 (all the mother liquors) were prepared by dropping circa 3 mL of each dispersion on TPU compression molded films. To ensure a uniform thickness for the coatings and in order to distribute them over the entire substrates, we made use of a brush to spread the dispersions (Figure 1). A similar amount (3 mL) of M.L.1, M.L.2 and M.L.5 was used to cast films of the respective materials, used to compare their antibacterial activity with the AgNP-based coating activities. A TPU film from M.L.6 was also prepared as a reference sample (Figure 1).

Another set of samples was prepared by using 3 mL of M.L.1, M.L.2, M.L.5 and M.L.6, respectively, and depositing them by a bar coater (TQC Sheen Automatic Film Applicator, Capelle aan den Ijssel, The Netherlands). TPU-based coatings were deposited at a rate of 50 mm/s with a “wet” thickness of 100 μm. A slower deposition rate (15 mm/s) was used for WPU-based coatings imposing a temperature of 60 °C through the use of a heating plate due to the different viscosity of this system. The same amount of M.L.1 and M.L.5 was also bar coated imposing a “wet” thickness of 200 μm. The curing of the coated substrates was carried out at room temperature at least for 4 h (Figure 1).

### 2.4. Characterization

The molecular, structural, thermal, mechanical and rheological properties together with surface morphology characteristics of the TPU have been widely reported in our previous work [12].

UV absorption spectra as a function of exposure time to ultraviolet light (curves taken every 15 s of exposure) were recorded using a PerkinElmer (Waltham, MA, USA) Lambda 900 spectrometer, for two samples at different Ag precursor concentration (25 mM and 50 mM). UV curing was carried out using a BlueWave 200 UV Light-Curing Spot Lamp at 4500 mW/cm^2^ (Hamamatsu Photonics, Arese, Italy).

UV Absorption spectra as a function of exposure time to sun light were recorded using a PerkinElmer (Waltham, MA, USA) Lambda 900 spectrometer, for a M.L.1 dispersion using a quartz cell and for a casted film prepared by the M.L.5 dispersion.

Contact angle (CA) measurements were performed using a CAM 200 from KSV Instruments Ltd. (Helsinky, Finland). Typically, a 10 μL drop of water was deposited on the surface of each prepared film to determine the static contact angles, respectively.

To capture high-resolution images and obtain elemental maps of the atomic elements of the different film and coatings, a scanning electron microscope (SEM) Zeiss EVO-MA10 (Carl Zeiss, Oberkochen, Germany) coupled to an energy dispersive X-ray spectroscopy (EDS) detector (X-max 50 mm^2^, Oxford Instruments, Oxford, UK) was utilized. Acceleration voltage used was 20 kV. Samples were only gold sputtered for SEM images acquisition.

### 2.5. Antibacterial Tests

#### 2.5.1. Bacterial Strains and Culture Conditions

The microorganisms used were *Escherichia coli* ATCC 25922 (*E. coli*) and *Staphylococcus aureus* ATCC 25923 (*S. aureus*), kindly supplied by R. Migliavacca (Department of Clinical Surgical, Diagnostic and Pediatric Sciences, University of Pavia, Italy). Bacteria were grown in their appropriate medium, overnight, under aerobic conditions at 37 °C using a shaker incubator (VDRL Stirrer 711/CT, Asal Srl, Italy): *E. coli* was inoculated in Luria Bertani broth (LB) (ForMedium^TM^, Hunstanton, Norfolk, UK) whereas *S. aureus* in BHI (Brain Heart Infusion broth) (Scharlab S.L., Sentmenat, Barcelon, Spain). Both cultures were reduced to a final density of 1 × 10^10^ cells/mL as determined by comparing the optical density (OD600) of the sample with a standard curve relating OD600 to cell number [28].

#### 2.5.2. Bacterial Viability

Materials were sterilized with ethanol 70%, washed twice in sterile ddH_2_O and incubated with 1 × 10^5^/sample. The inoculation was carried out for direct contact and indirect contact. The viability was assessed with 3-(4,5-dimethylthiazol-2-yl)-2,5-diphenyltetrazolium bromide (MTT) colorimetric assay (Sigma-Aldrich, St. Louis, SM, USA) on bacteria in direct contact and on bacterial suspension in indirect contact. After 3 h of incubation, at 37 °C, the MTT reaction was stopped by adding solution C (2-propanol, HCl 0.04 N), and further incubated for 15 min at 37 °C. The colorimetric reaction was analyzed at CLARIOstar (BMG Labtech, Ortenberg, Germany) at 570 nm wavelength with 630 nm as reference wavelength. Results firstly normalized to Tissue Culture Plate (TCP) and then to TPU set as 100%. The experiment was carried out in triplicate and repeated 2 times.

##### Direct Contact

The experiment was performed on (I) planktonic bacterial cultures and (II) directly on bacterial adhesion on materials, at 24 h of incubation. 200 µL of 1 × 10^5^ were inoculated onto materials, and in TCP used as the positive control, contained into 96-well flat-bottomed sterile polystyrene microplates (Euroclone S.p.a, Milan, Italy), incubated at 37 °C.

##### Indirect Contact

LB broth was inoculated on materials contained in a 96-well flat-bottomed sterile microplate, incubated overnight at 37 °C. Serial dilutions of the inoculated solution were performed starting from a volume of 100 µL of solution. 100 µL of 1 × 10^5^ of bacteria were inoculated and incubated overnight, at 37 °C.

#### 2.5.3. SEM Analysis

Bacteria were incubated on TPU polymeric films for 24 h at 37 °C. The films were washed carefully with phosphate buffer solution (PBS) 1X and fixed with 2.5% (*v*/*v*) glutaraldehyde (Sigma-Aldrich, St. Louis, SM, USA) in 0.1 M Na-cacodylate buffer (Sigma-Aldrich, St. Louis, SM, USA) (pH 7.2), for 1 h at 4 °C. After two washes with Na-cacodylate, to remove excess of glutaraldehyde, *S. aureus* samples were dehydrated using increasing concentrations of ethanol (25, 50, 75%) for 5 min and two washes of 96% ethanol for 10 min. *E. coli* samples were dehydrated just with two washes of 96% ethanol (Merck Life Science S.r.l., Milano, Italy) for 10 min. The samples were lyophilized for 3 h using a K-850 apparatus (Emitech Ltd, Ashford, UK) and placed on a mounting base. Finally, TPU films were sputter coated with gold and investigated using a Zeiss EVO-MA10 scanning electron microscope (Carl Zeiss, Oberkochen, Germany), 20 kV acceleration voltage.

#### 2.5.4. Statistical Analysis

All the statistical calculations related to antibacterial tests were carried out using GraphPad Prism 5.0 (GraphPad Inc., San Diego, CA, USA). Statistical analysis was performed using Student’s unpaired, two-sided *t*-test (significance level of *p* ≤ 0.05).

### 2.6. Adhesion Tests

The bond strength measurements were performed using a rotational rheometer AR 2000 from TA Instruments (New Castle, DE, USA) in parallel-plate geometry. The load cell has a force resolution of 1 mN, while the stepper motor can resolve distances as small as 0.001 mm. All the experiments were performed at 10 °C by using a Peltier temperature controller (TA Instruments New Castle, DE, USA). The experiment consisted of two steps: (1) a compression with upper plate moving at a constant velocity (0.1 mm/s) in the downward direction, (2) at the fixed gap (0.4 mm), the motion of the upper plate is reversed at the same speed, leading to a position sufficient to guarantee cohesive failure.

## 3. Results

### 3.1. Preparation of the Mother Liquors

Six M.L.s were prepared with the three different antibacterial agents under investigation, i.e., silver, titanium dioxide and chitosan (Figure 1), following the procedure reported above (see Materials and Methods, Section 2.2).

### 3.2. Silver Nanoparticles Formation

**Figure 2:**
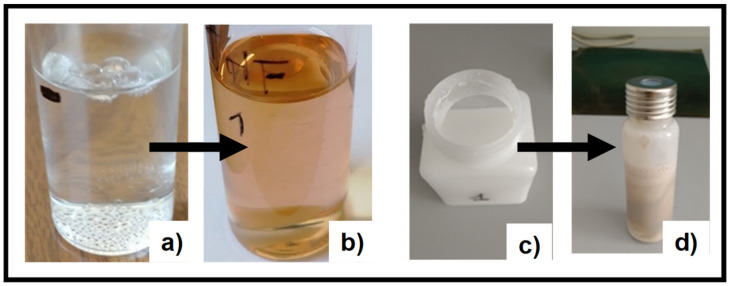
Photographic image of M.L.1 (TPU-AgNP-1) and M.L.5 (WPU-AgNP) before (**a**,**c**) and after (**b**,**d**) sun light exposition. M.L.: mother liquors.

Silver nanoparticle (AgNP) dispersions were prepared from TPU solutions in THF, adding a known amount of an AgNO_3_ aqueous solution. AgNO_3_ water-based solutions were prepared at two different concentrations (5 and 25 mM) and used to prepare two different AgNP/TPU dispersions, denoted as M.L.2 and M.L.1 in Figure 1, respectively. A qualitative indication of nanoparticle formation was registered after exposing the prepared dispersions at sun light. A variation from a transparent/whitish dispersion to a pink/yellow/brown sample, as a consequence of some light exposure (30–60 min) or temperature variation, was observed (Figure 2a,b). 

UV spectra collected for M.L.1 after controlled exposure to sun light and the relative photographic images of the dispersion are reported in Figure S2. These experiments were performed on liquid sample and suggest that after 2 h a relevant part of silver is already reduced when the solution started already to have a pinkish colour.

WPUs are polyurethanes provided with hydrophilic properties, which make them dispersible in water preventing the use of volatile organic compounds (VOC) for their dissolution [26]. Taking advantage of their properties, we promoted AgNP formation by using a similar procedure and adding a small amount of AgNO_3_ aqueous solution to a WPU aqueous dispersion denoted as M.L.5 in Figure 1. As a result of the nanoparticles formation, a colour change from the initial white (WPU dispersion) to a brownish final dispersion (WPU-AgNP) was observed (Figure 2c,d).

**Figure 3:**
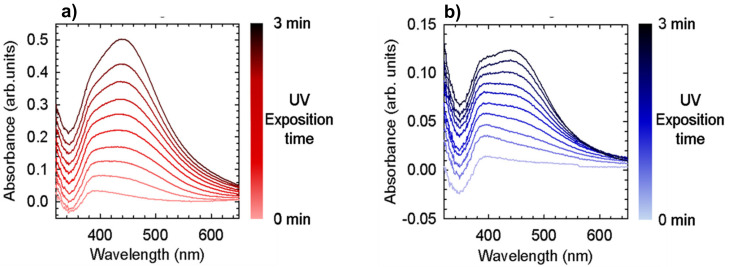
UV Absorption spectra as a function of exposure time to ultraviolet light (curves taken every 15 s of exposure) for two samples at different Ag precursor concentration, 50 mM red graph (**a**) and 25 mM blue graph (**b**).

To study the AgNP formation, two films were prepared, starting from a solution of 60 mg/mL TPU + 50 mM AgNO_3_ and 60 mg/mL TPU + 25 mM AgNO_3_, respectively. Reducing by one/third their total concentrations by THF addition, the two diluted solutions were deposited by spin-coating, obtaining homogeneous films with a thickness of about 200 nm (measured by a profilometer). In Figure 3, UV absorption spectra at different acquisition time of the films obtained from solutions at 50 mM (Figure 3a) and at 25 mM concentration of silver precursor concentration (Figure 3b) are presented. These samples were then progressively irradiated with ultraviolet light for short intervals (15 s) and an absorption spectrum was collected after each treatment. Figure 3 shows that the exposure to UV light causes an increase in the absorption band at about 450 nm, which is proof of the progressive formation of AgNP within the film. The characteristic surface plasmon resonance band of Ag nanoparticles is registered and its intensity depends upon the Ag precursor concentration. The highest Ag precursor concentration yields the highest absorbance features (Figure 3a). Moreover, by triggering the NP formation reaction by controlled UV exposition time, an increased content of Ag nanoparticles is promoted independently from the initial Ag concentration as result of the successful AgNP formation. A similar experiment with an extra UV absorption spectrum collected after 24 h was performed and reported in Figure S3. No significant differences between the spectra collected after 3 min and 24 h were observed and therefore we concluded that the reaction is almost complete within minute-scale under these conditions.

UV absorption spectra were also collected for a M.L.5 film prepared using the spin coater according to the procedure reported above. A comparison between two spectra collected just after the deposition and after 2 h of sun light exposure are presented in Figure S4. An obvious peak associated to the AgNP formation is visible for the sample subjected to sun light exposure.

The wettability of AgNP-TPU-Film1 and AgNP-WPU-Film was investigated and compared to that of TPU-Film and a casted film from a WPU dispersion, performing static CA measurements using water. The equilibrium contact angle of a drop of water on an ideal surface conventionally quantifies the wettability of a solid by a liquid. From the CA data collected, it is possible to observe the predominantly hydrophobic nature of the AgNP-TPU-Film1 (ca. 115°) compared to TPU-Film (ca. 95°). Hydrophilic qualities (ca. 67°) were observed for the film from a WPU dispersion, and a slight increase in the CA value is registered after the introduction of AgNP (about 78° for AgNP-WPU-Film).

**Figure 4:**
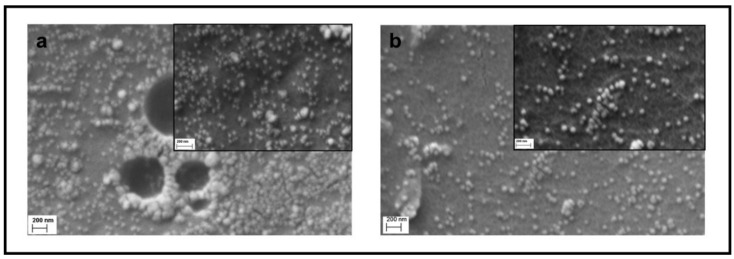
Surface morphology images of films prepared by M.L.1 (**a**) and M.L.5 (**b**) at 76 kX. The inset SEM images were collected at 148 kX.

SEM measurements were carried out to further investigate the presence of fillers on the surface of film prepared by drop casting an equivalent amount of M.L.1 and M.L.5, respectively. Two SEM images of each sample, taken at different magnifications (76 kX and 148 kX) are reported in Figure 4; AgNP of spherical morphology and dimensions ranging from 20 to 50 nm are observed. They appear to be abundant and well dispersed on the polymer matrix, supporting UV data on AgNP formation. Similarly, a homogeneous film obtained from M.L.1 and deposited by spin-coating, was subjected to SEM investigations, after a plasma treatment (plasma oxigen 5 min, 43W). The micrographs reported in Figure S5, confirm the morphology and the dimensions of the AgNP.

### 3.3. Preparation of the Coatings

Coatings of all the six M.L.s were prepared on TPU substrates using a fixed amount of each M.L. and a brush (Figure 1). An average thickness of 50 µm was measured with a profilometer for coatings obtained using this deposition techniques.

From the antibacterial properties observed (see Section 3.5) M.L.1, M.L.2, M.L.5 and M.L.6, were selected to be deposited via bar coater, using a bar of 100 µm (Figure 1). This allowed the comparison among the thin layer bar coatings characterized by a different silver precursor concentration, as in the case of AgNP-TPU-Bar1 and AgNP-TPU-Bar3, and that formed in a WPU dispersion (AgNP-WPU-Bar1). A TPU based coating from M.L.6 was similarly deposited via bar coating and used as a reference for the evaluation of the coating morphology. These coatings presented an average thickness of 10–20 µm. The two most promising coatings characterized by the highest concentration of the silver precursor in water (25 mM), were deposited using a bar of 200 µm (Figure 1). These samples, denoted as AgNP-TPU-Bar2 and AgNP-WPU-Bar2, presented a final average thickness of 50 µm.

Finally, to evaluate the effect of a different precursor concentration and the nanoparticle formation in different systems, AgNP-TPU-Film1, AgNP-TPU-Film2 and AgNP-WPU-Film were prepared and compared to the reference TPU-Film (Figure 1).

### 3.4. Film and Coatings Morphology

**Figure 5:**
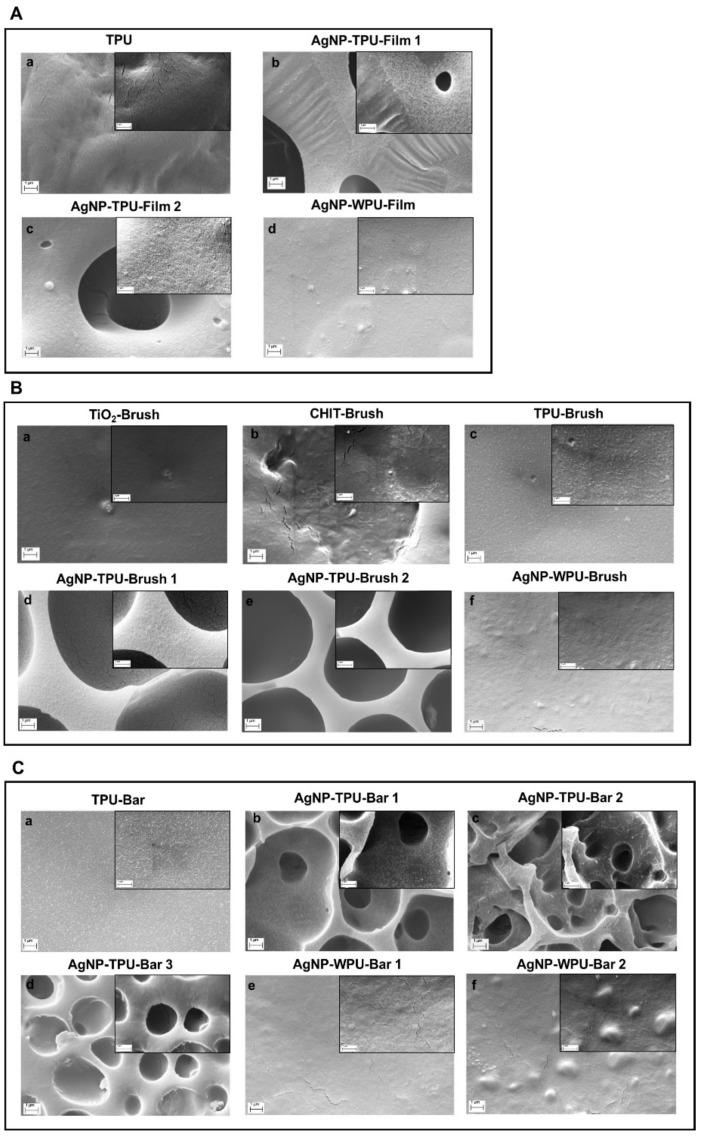
SEM images of thermoplastic polyurethane (TPU) and waterborne polyurethane (WPU) polymeric films at 20 kX (scale bar 1 μm), insert in the right corner at 40 kX (scale bar 1 μm). Panel (**A**): TPU and casted films; panel (**B**): brush films; panel (**C**): bar coater films.

Film morphology and quality of the coating deposition were evaluated by examining several different areas of the prepared samples using SEM investigation. SEM micrographs of TPU film and casted films containing AgNP are reported in Figure 5A. Beside the homogeneity and regularity of the TPU (Figure 5A(a)) prepared via THF dissolution and solvent casting and AgNP-WPU film (Figure 5A(d)) prepared via solvent casting of the corresponding water based dispersion, more interesting morphologies are observed for AgNP-TPU-Film1 and AgNP-TPU-Film2 (Figure 5A(b,c)). A microporous cell morphology, characterized by an average pore size of about 10 μm, is observed. This morphology is independent from the Ag precursor concentration on condition that film is prepared starting from a ternary system of polymer/solvent/nonsolvent under certain conditions, as reported in literature [29]. The microporous morphology is observed even if coatings are prepared from the same M.L. and different deposition techniques are used (Figure 5, Panels B and C). In particular, AgNP-TPU-Brush1 and AgNP-TPU-Brush2 are characterized by microporous morphology (Figure 5B(d,e)). Further examples of porous morphology are reported at different magnifications in Figures S6 and S7, respectively. Similar results are observed for the bar coated films AgNP-TPU-Bar1, AgNP-TPU-Bar2 and AgNP-TPU-Bar3 (Figure 5C(b–d)), which present exactly the same morphology.

On the contrary, TPU and WPU-AgNP materials, independently from the deposition technique employed, present flat and homogenous surfaces (Figure 5B(c,f),C(a,e,f)). Finally, TiO_2_-Brush and CHIT-Brush show a flat and quite homogeneous surface (Figure 5B(a,b)) similar to TPU and WPU-based samples.

**Figure 6:**
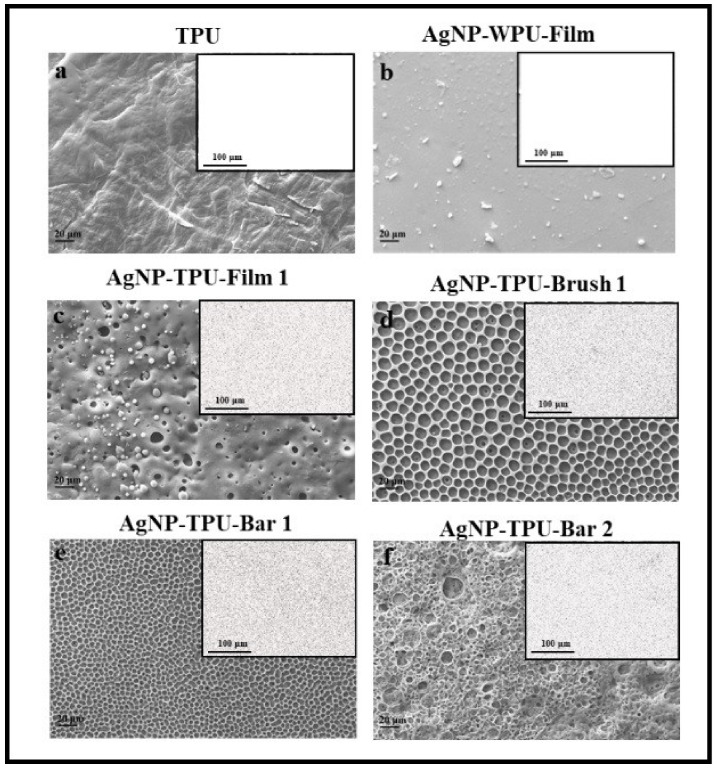
SEM images of TPU and WPU polymeric films at 1kX (scale bar 20 μm). Insert in the right corner the EDS map (scale bar 100 μm). TPU (**a**), AgNP-WPU-Film (**b**), AgNP-TPU-Film1 (**c**), AgNP-TPU-Brush1 (**d**), AgNP-TPU-Bar1 (**e**) and AgNP-TPU-Bar2 (**f**).

EDS analysis was performed on all the samples prepared. Here, we report SEM images and the relative EDS maps of the materials deriving from M.L.1 that show the most interesting antibacterial activities (as presented in the next paragraph), as compared to those of TPU and AgNP-WPU films (Figure 6). The porous morphology allows to detect AgNP presence although all these samples are characterized by a very low concentration of the silver precursor. It follows that a clear presence of AgNP is observed for AgNP-TPU-Film1, AgNP-TPU-Brush1, AgNP-TPU-Bar1 and AgNP-TPU-Bar2 (Figure 6c–f, respectively). TPU film does not show any trace of silver similarly to AgNP-WPU-Film for which silver nanoparticle presence was expected, as shown in Figure 4b. The sterilization procedure has an influence on the presence of nanoparticles on film surfaces, especially when WPU is used to prepare AgNP-containing materials. More details will be given in the Section 4.

### 3.5. Antibacterial Properties

**Figure 7:**
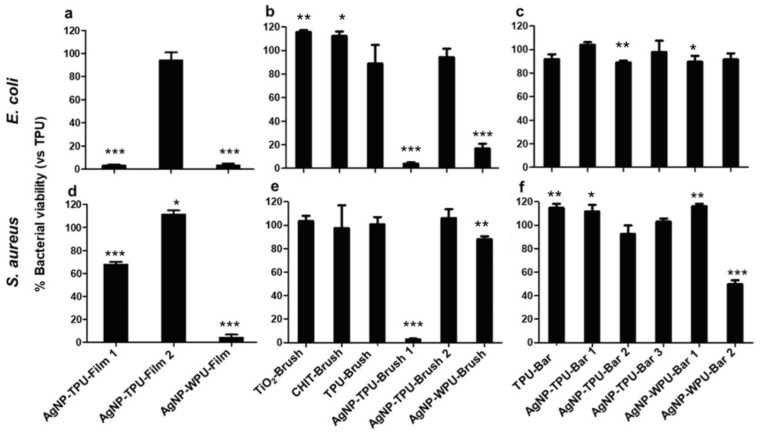
Cell viability on planktonic *E. coli* (**a**–**c**) and *S. aureus* (**d**–**f**) cultures after their removal from polymeric films (direct contact). After 24 h of incubation with TPU and WPU-deposited materials, both bacterial cultures were removed and cell viability evaluated by 3-(4,5-dimethylthiazol-2-yl)-2,5-diphenyltetrazolium bromide (MTT) test. The figure shows the percentage of viability of all 15 samples related to TPU set as 100%. Bars indicate mean values ± SD of the mean of results from two experiments. *- Student’s *t*-test, statistical significance values were *p* < 0.05 (*), *p* < 0.01 (**) and *p* < 0.001 (***).

**Figure 8:**
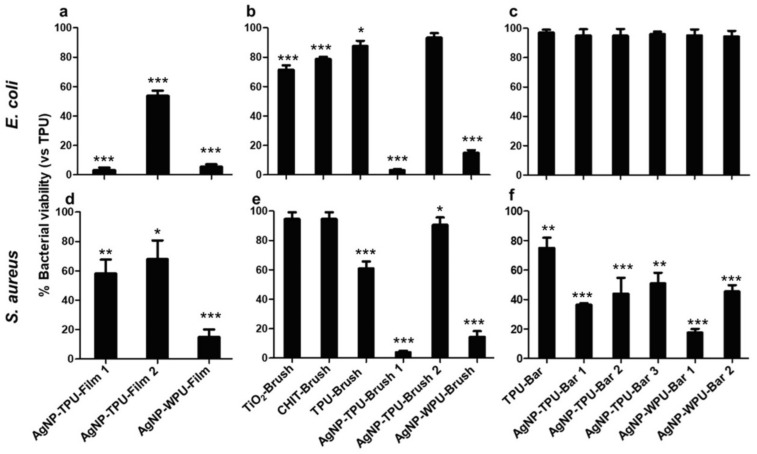
Adherent cell viability of *E. coli* (**a**–**c**) and *S. aureus* (**d**–**f**) cultures on polymeric films. After 24 h of incubation with TPU and WPU-deposited materials, both bacterial cultures were removed and cell viability evaluated by an MTT test. The figure shows the percentage of viability of all 15 samples related to TPU set as 100%. Bars indicate mean values ± SD of the mean of results from two experiments. *- Student’s *t*-test, statistical significance values were *p* < 0.05 (*) and *p* < 0.01 (**), and *p* < 0.001 (***).

The antimicrobial tests of polymeric films were performed through the MTT colorimetric assay. The viability was investigated through direct contact and indirect contact at 24 h. In direct contact, the antibacterial test was carried out both on planktonic bacterial cultures after their removal from the films (Figure 7) and directly onto the polymeric films (Figure 8). 

We evaluated the viability on planktonic culture and adhesive bacteria even in a short time of incubation, 6 h, to check whether an antibacterial effect was present. These results are reported in Supplementary Materials (Figures S8 and S9). In Figure 7 viability data on planktonic bacterial cultures allow for a comparison between *E. coli* (a–c) and *S. aureus* (d–f) according to the deposition method on PU: solvent casted film (a, d), brush (b, e) and bar coater (c, f). *E. coli* inhibition is observed for AgNP-TPU-Film1 (96.5%), AgNP-WPU-Film (95.5%), AgNP-TPU-Brush1 (96%) and AgNP-WPU-Brush (84.5%). *S. aureus* is inhibited by AgNP-WPU-Film (93.7%), AgNP-TPU-Brush1 (92.7%), whereas AgNP-TPU-Film1 decreases the staphylococcal proliferation of 30% and AgNP-WPU-Bar2 of 41%. Among the bar coated samples, only AgNP-WPU-Bar2 showed less reduction in Gram-positive. However, data obtained after 6 h (Figure S8) and 24 h (Figure 7) are interesting to compare because some films have an immediate antibacterial property compared to the incubation for a long time. In particular, all of the samples result as active in Gram-positive (Figure S8d–f), with the exception of AgNP-WPU-Brush. Moreover, the films that retain their antibacterial properties against gram negative *E. coli*, after 6 h and 24 h, are the solvent casted films, whereas AgNP-TPU-Brush1 is effective after a long incubation time versus both bacterial strains (Figure 7).

The viability related to adhesion after 24 h, is reported in Figure 8. The samples that show an inhibitory activity on *E. coli* adhesion are not different from those effective in planktonic culture; in addition, AgNP-TPU-Film2, TiO_2_-Brush and CHIT-Brush showed a reduction of 46%, 30% and 20%, respectively. The adhesion of *S. aureus* is still reduced by the same polymeric films effective in planktonic culture, even though AgNP-WPU-Film is more efficient against planktonic culture. Moreover, other films showed an interesting result: AgNP-WPU-Brush reported only ~15% of viability compared to 80% in planktonic bacteria and bar coated films are efficient in adhesion, particularly AgNP-WPU-Bar1.

The anti-adhesive efficacy of films in Gram-positive is more evident in the short term, as reported in Figure S9d–f, whereas the effect on *E. coli* is similar between, 6 h (Figure S9a,b) and 24 h (Figure 8a,b). Bar coated films in Gram-negative show a different trend, all of them being more effective at 6 h (Figure S9c) than at 24 h (Figure 8c), namely AgNP-TPU-Bar 1, AgNP-TPU-Bar 2, AgNP-WPU-Bar 1 and AgNP-WPU-Bar 2.

**Figure 9:**
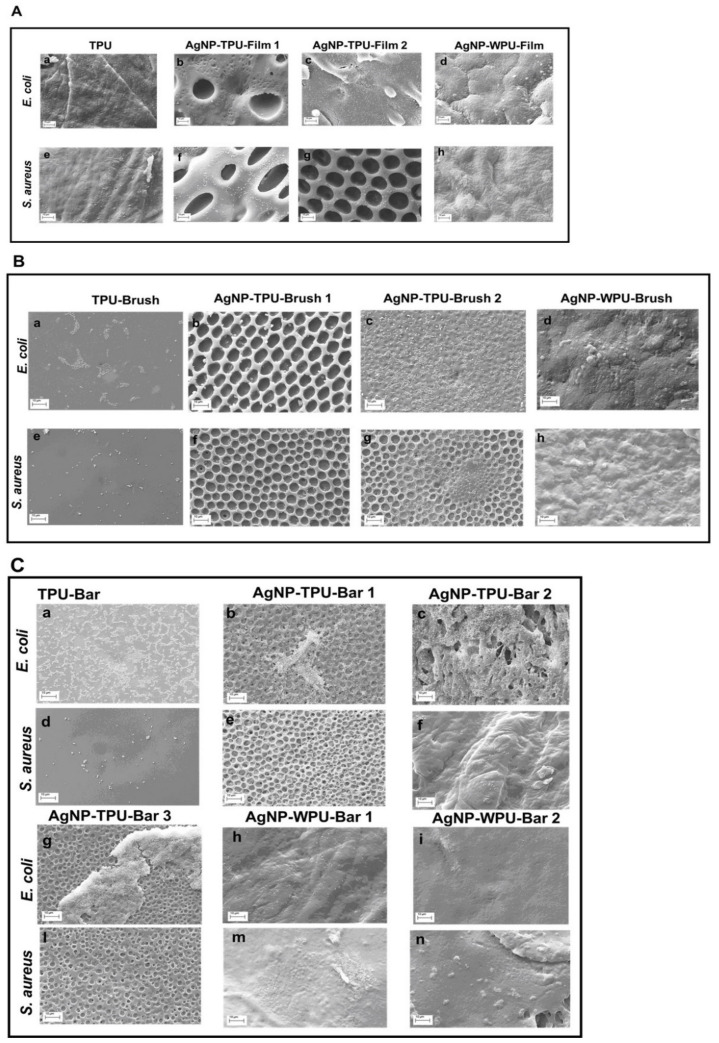
SEM images of bacteria adherent on TPU and WPU polymeric films at 6 kX (scale bar 10 μm). Panel (**A**): *E. coli* (**a**,**d**), *S. aureus* (**e**–**h**) on TPU and casted films; panel (**B**): *E. coli* (**a**,**d**), *S. aureus* (**e**–**h**) on brush films; panel (**C**): *E. coli* (**a**–**c**; **g**–**i**), *S. aureus* (**d**–**f**; **l**–**n**) on bar coater films.

The data obtained from bacterial adhesion (after 24 h) are supported by SEM analysis (Figure 9 and  Figure S10). The figure compares *E. coli* and *S. aureus* adherent on casted films (A), brush films (B) and bar coated films (C). In panel A, it is interesting to note that Gram-negative bacteria are few and dispersed on the surface, whereas in AgNP-WPU-Film they are concentrated in few spots. In addition, AgNP-WPU-Film is the only casted film that can reduce the adhesion of Gram-positive *S. aureus.* Indeed, these bacteria are present in spots like *E. coli* but with the difference that there are less concentrated spots. Panel B shows the comparison between films deposited through brush. The most efficient inhibiting samples are AgNP-TPU-Brush1 and AgNP-WPU-Brush both in Gram-negative and Gram-positive. It is interesting to see that bacteria, in AgNP-TPU-Brush1, are localized inside the holes which characterize the film. The efficiency of this material is high in both *E. coli* and *S. aureus*. The great adhesion of both bacterial strains on AgNP-TPU-Brush2 is supported not only by the low Ag concentration (5 mM), but even for alkaline pH (8). A high pH value is involved in the high proliferation and adhesion of bacteria. All the other films, instead, had a neutral pH (Table S1). In AgNP-WPU-Brush bacteria are immersed in the matrix. Lastly, bar coated films, as shown in panel C, have anti-adhesive properties just for *S. aureus.*

**Figure 10:**
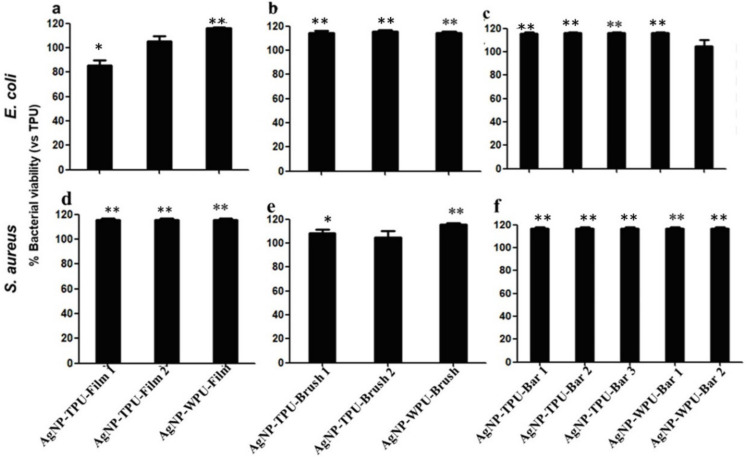
Bacterial viability after incubation with Ag released in LB. TPU-films were incubated overnight with Luria Bertani (LB) to release Ag. The solutions, after their removal from films, were incubated with *E. coli* (**a**–**c**) and *S. aureus* (**d**–**f**). Cell viability was evaluated by MTT test. The figure shows the percentage of viability of all 11 samples related to TPU set as 100%. *- Student’s *t*-test, statistical significance values were *p* < 0.05 (*) and *p* < 0.01 (**).

Bacterial viability has also been evaluated, on TPU-films incubated overnight with LB broth to release Ag. This method, called indirect because bacteria are not in contact with the films, has been performed to determine the inhibitory effect of silver released in the solution. The results are reported in Figure 10. The only solution that slightly reduced the viability of *E. coli* was AgNP-TPU-Film1, while the solutions had no effect against *S. aureus.*

### 3.6. Adhesion Test

**Figure 11:**
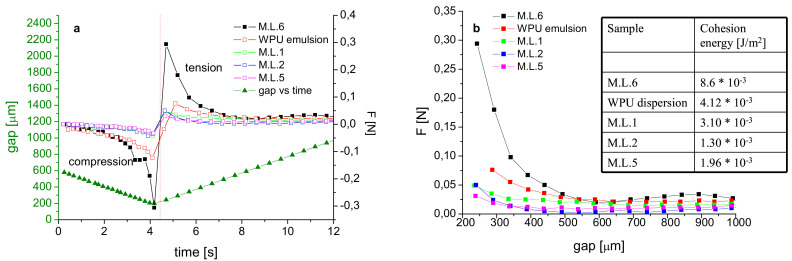
Normal force and gap as a function of time, registered in both compression and tension steps (**a**). Normal force as function of gap in the tension experiment (**b**). The inset table in (**b**) reports the cohesion energies.

To study the cohesive energy of the prepared coatings, we investigated their fluid behavior with the idea of relating the measured separation force of the fluid directly to their adhesive performance [30,31]. For this reason, bond strength measurements were performed on the Ag-containing M.L.1, M.L.2, M.L.5 and compared to M.L.6 and a WPU dispersion. The fluid to test was placed between the two disks (25 mm parallel plates) completely filling the gap, and the temperature was kept at 10 °C. The experiment consists of a compression step, associated with a sample squeeze and generating an opposing force registered by the instrument load cell, and a tension step, leading to a position sufficient to guarantee cohesive failure and associated with a positive tensile force related to the cohesive force of the fluid. Cohesive failure in fact is the only failure we registered at the end of each experiment. The complete experiment covering both compression and tension is presented in Figure 11a. The maximum force registered in compression is ~−0.3 N and ~0.3 N in tension for M.L.6; whereas WPU dispersion presents values of a maximum force of ~−0.1 N and ~0.1 N in compression and tension, respectively. For Ag-containing samples a significant reduction in the maximum force values is observed. Since our objective is to measure the separation force as a function of gap, Figure 11b reports the trend of the normal force as a function of gap registered in the tension step. The calculated cohesive energies are reported in the inset of Figure 11b and show the differences between TPU based and WPU based M.L. and the effect ascribed to AgNP presence.

## 4. Discussion

As in our previous work [12], we elected to use silver, titanium dioxide and chitosan for their recognized antibacterial properties. In the present study, however, the use of these compounds in the form of coatings is presented with the idea of providing a direct comparison with alternative preparation techniques (i.e., melt processing and post-processing). For this reason, different antibacterial M.L.s were prepared and used for the preparation of film and coatings (Figure 1). Besides the solvent casted films, which turned out to be very efficient and good reference samples, coatings from the different M.L.s were prepared on TPU substrates by using a brush in the first set of experiments and a film applicator successively. The bar coater was used with the idea of allowing a more homogeneous deposition of the coating. However, coatings with a thickness of 10–20 μm were obtained and only the employment of a 200 μm bar for some selected samples allowed to obtain thicker films comparable with the brushed one (~50 μm). For M.L.3 and 4 we used the same antibacterial materials, simply dissolved or dispersed in an opportune liquid, a different strategy was employed for the silver-based film and coatings.

We used a silver salt precursor to promote nanoparticle formation and create more efficient antibacterial materials with less possible cytotoxicity problems caused by silver release. This comes from the unique properties of metal nanoparticles, due to their high surface/volume ratio. It is in this sense that, silver nanoparticles, in view of their “easy” preparation via economic and sustainable methods, are becoming more interesting especially for use in biomedical applications. Several preparation methods have been reported [24,32,33,34,35]. These preparation methods are carried out using a series of components whose control allows to influence the size and shape of the AgNP. Usually, this occurs through silver(I) precursor reduction and consists of two steps: nucleation and growth. The reduction requires the use of a reducing agent capable of promoting silver metallic formation. Since the metal form is not thermodynamically stable, especially in aqueous or alcoholic solution used, a stabilizer is used to prevent a further oxidation of the obtained metal. Stabilizers not only contrast the high reduction potential of silver protecting the nanoparticles but prevent agglomeration and influence their size and shape, playing a determinant role in the nucleation and growth stages. For this reason, several different methods have been developed to examine not only different reducing agents, but also several stabilizers [24].

In our study, we introduced TPU as reducing agent and stabilizer. The electron rich groups (i.e., carbonilic, carbossilic, amminic and aromatic groups) constituting the TPU polymer chain serve as reducing agents to promote silver precursor reduction. The polymeric nature of TPU ensures the stabilizing effect for nanoparticle stability. The observed colour variation of M.L.1, 2 and 5 after sunlight exposition (Figure 2) and the characteristic surface plasmon resonance band of Ag nanoparticles (Figure 3), whose intensity depends on silver precursor concentration and UV exposition, confirm the nanoparticle formation. To the best of our knowledge, this is the first time that silver nanoparticle formation has occurred through the use of a single component which is able to guarantee both functions. We have only found a work where a similar strategy was utilized, in particular N,N-dimethylformamide (DMF) was used as a solvent and reducing agent, and polyurethane as a structure-directing agent [36]. However, we successfully used this strategy with other polymeric systems having similar required characteristics but dispersed or dissolved in different solvents (i.e., WPU and chitosan, see also Figure S11), showing how AgNP formation is independent from the solvent employed. Here, we only report a comparison with a WPU, highlighting the potentiality sustainability of this method. Furthermore, even in this process where a single component is used for AgNP formation, the shape and size of the nanoparticles can be triggered and controlled by modulating the exposure to light or temperature. Silver nanoparticles, with an average dimension of ~20–50 nm, have been observed for the different systems considered. As observed in the SEM images reported in this paper, their presence and dispersion can be affected by the precursor concentration and the deposition technique. EDS maps of the samples confirm these data, highlighting the role played by the porous morphology in AgNP detection (Figure 6). The low concentration of the silver precursor allows to detect AgNP presence only for porous materials, where the amount of detectable silver is inevitably higher. AgNP presence, also expected for WPU-Film is not always observed. In particular, we manage to highlight, by SEM (Figure 4b), the presence of silver nanoparticles over the film prepared by M.L.5, avoiding the sterilization, whereas the low concentration of the silver precursor prevents their detection by EDS (Figure 6b), in the absence of porous morphology. Finally, the versatility of this preparation method could promote the use of AgNP in many fields of applications, especially those for which a well dispersed and low concentrated amount of nanoparticles is requested [37].

The film morphology and the observed porosity derive from the choice of the polymer and the solvents used for M.L. preparation. Many methods for the preparation of porous structures have been used and reported in the literature, from electrostatic spinning to laser ablation techniques or gas flow [38,39,40]. However, one of the most used and interesting is the phase-inversion process, a method where a homogeneous polymer-solution is put into contact with a nonsolvent, resulting in phase separation responsible of the porous formation [29,41]. The separation into a polymer-rich phase and polymer-poor phase is guaranteed by the exchange of solvent/nonsolvent at the interface. The final morphology of the film obtained is regulated by several parameters correlated to the kinetics and the thermodynamics of the phase separation and therefore many equilibrium phase diagrams for different relevant systems were built as a useful tool for morphology control [29,42].

Two different phase separation processes have been observed and are competitive during membrane formation: solid–liquid (S–L) demixing and liquid–liquid (L–L) demixing. (S–L) demixing, or crystallization, leads to membranes with a spherulitic or axialitic morphology, whereas (L–L) demixing to porous structure [29]. At lower polymer concentrations the typical cellular morphology is observed which is a result of a diffusion-induced phase separation process typical of (L–L) demixing [29,42]. Similarly in our AgNP-TPU systems, being at low polymer concentration (5.6 wt %) with about 9 wt % of water and neglecting AgNO_3_ content (0.04 wt %), a porous morphology was obtained for our “ternary system” independently from the casting or deposition technique employed. Differently from what is reported in the literature, where an immersion precipitation process through the use of a coagulant bath is used until the exchange of solvent and nonsolvent is completed, our system consists of a one pot preparation and only after the film is casted or deposited [42,43,44]. Moreover, the homogeneous and abundant AgNP presence observed after plasma treatment on the film obtained from M.L.1 and deposited by spin-coating (Figure S5), confirms that the AgNO_3_ aggregates solvated in the polymer matrix during the solidification and react with the proper TPU functional groups to be reduced in metallic silver while the volatile THF rapidly evaporated [43,45]. Where this porosity is not required, it is always possible to limit the use of the nonsolvent as in the case of WPU (only water used) or to simply dissolve the silver precursor directly in THF, the solvent employed for TPU dissolution.

Several antibacterial tests were carried out through direct contact and indirect contact, giving information on the viability of planktonic culture and adhesion of bacteria at two different times of incubation, 6 h and 24 h, respectively (Figures S8, S9, Figure 7and Figure 8). Bacterial planktonic culture tests at 24 h, show better performances for the AgNP casted films, with the highest precursor concentration versus both bacterial strains. A similar result is also observed for the brushed coating (AgNP-TPU-Brush1), clearly demonstrating the role played by the initial precursor concentration. Bar coated samples, even those characterized by the highest amount of silver, do not present similar activities. This suggests a probable loss or a lower transferred amount of the antibacterial during this deposition procedure and for this reason, only at shorter times (6 h) did their capability resist against *S. aureus* in almost all the samples, while the casted films are also active versus *E. coli*. An analogous trend is also observed in our analysis of the bacterial adhesion tests at 6 h (Figure S9), where all the samples show good properties mainly versus *S. aureus*, with the exception of the casted films which were once again active against both strains. On the contrary, adhesion tests at 24 h show a general improvement of antibacterial properties compared to the viability on planktonic culture at 24 h, especially against *S. aureus*, with these activities being directly measured on the film surfaces (Figure 8). These data are supported by the SEM analysis performed to evaluate bacterial adhesion of *E. coli* and *S. aureus* on TPU and casted films (A); on brush films (B) and bar coated films (C) (Figure 9).

To assess whether these materials could present some release of the AgNP, bacterial viability tests after incubation with Ag released in LB were performed. Since all these materials exhibit very high viability values, associated with low antibacterial activity, we conclude that these systems do not release nanoparticles easily.

By playing with the solvent/polymer system and concentrations, important processing and deposition parameters, such as viscosity and wettability, can be influenced, allowing the employment of several deposition techniques, not least the spray one. Some of the formulated antibacterial M.L.s, in particular AgNP-TPU and AgNP-WPU, have been successfully deposited via dip coating on parts of medical probes (Figure S12). Moreover, several substrates according to the final purpose can be used. For convenience, we deposited such coatings on TPU substrate in order to promote adhesion and aware of substrate surface deformations and stresses arising from the use of the same solvent. However, attempts were made on polyethylene (PE) substrates (Supporting Information, Figure S13) without success, since we could not properly functionalize the substrate surface [26]. This weakened the adhesion between the coating and substrate, denying the possibility of performing antibacterial test. However, a simple corona treatment on PE surfaces could enhance the adhesion and allows the preparation of antibacterial coatings over other interesting polymer substrates with commercial relevance.

In our experience, the adhesion of PU-based coatings can be affected by chemical structure modifications associated with reduced hydrogen bonding efficiency responsible for a limited capability of microphase separation [26]. Moreover, we recently reported on how the TPU hydrogen bonding efficiency can be perturbed by the use of silver, titanium dioxide and chitosan fillers with a consequent effect on the complex microphase separation of this material [12]. Since the same antibacterials were used for film and coatings preparation, we believe that the observed trend of tack test experiments is a result of the lower molecular ordering introduced. The significant reduction of the cohesion energies observed from M.L.6 to M.L.1 and M.L.2, and from WPU dispersion to M.L.5, respectively, supports this thesis and allows us to associate the reduced adhesion properties and the observed cohesive failures with the lower molecular ordering introduced by the presence of the antibacterial agents.

## 5. Conclusions

In this paper PU-based coatings and films were successfully prepared and investigated. A new simplified method allowed the preparation of AgNP-based materials, with effective antibacterial properties and starting from a very low amount of silver precursor. The results are promising since we only used TPU as the reducing agent and stabilizer for silver nanoparticles preparation. UV and SEM data support the formation of silver nanoparticles whose dispersion results to be quite homogeneous, as confirmed by EDS experiments, especially for the films characterized by a porous morphology.

Silver based mother liquors, obtained with a one pot preparation method, can be easily modified according to the intended deposition technique, allowing for the preparation of coatings with interesting properties for commercial applications. This concept is further strengthened if we consider that the concentrations of the silver precursor were deliberately chosen to be very low to show the potential of this method. Moreover, silver nanoparticle formation was obtained using the same procedure with different polymeric systems, characterized by similar electron rich functional groups. The aforementioned characteristics highlight the potential of these systems and their versatility. This versatility could broaden the application fields of AgNP and promote their use as materials for adhesives, paints, packaging, electronics, gas separation, catalysis, battery separators, water treatment and medicine, including chronic neurodegenerative diseases.

## Supplementary Materials

The following are available online at https://www.mdpi.com/1996-1944/13/19/4296/s1. Figure S1: Surface morphology images of a homogeneous film obtained from M.L.1 and deposited by spin-coating at different magnifications after plasma treatment; Figure S2. UV Absorption spectra as a function of exposure time to sun light for a M.L.1 sample; Figure S3. UV Absorption spectra as a function of exposure time to ultraviolet light for a samples at 50 mM Ag precursor concentration; Figure S4. UV Absorption spectra as a function of exposure time to sun light for a film of M.L.5 prepared by spin coating deposition; Figure S5. Surface morphology images of a homogeneous film obtained from M.L.1 and deposited by spin-coating at different magnifications after plasma treatment. Figure S6: Photographic image and surface morphology images of AgNP-TPU-Film1 at different magnifications; Figure S7: Photographic image and surface morphology images of AgNP-TPU-Brush1 at different magnifications; Figure S8: Planktonic bacterial viability through direct contact with materials. Comparison of *E. coli* ATCC 25922 and *S. aureus* ATCC 25923 viability after 6 h of incubation; Figure S9: Bacterial adhesion on films through direct contact with materials. Comparison of *E. coli* ATCC 25922 and *S. aureus* ATCC 25923 viability after 6 h of incubation; Table S1. pH values of the different reported samples after 24 h at 37 °C measured in physiologic solution and Luria Bertani broth; Figure S10. SEM images of bacteria adherent on TPU and WPU polymeric films at 3 kX (scale bar 10 μm). Figure S11: Photographic image of AgNP dispersed in a CHIT-solution after sun light exposition; Figure S12: Prototypes of biomedical probe in polyurethane coated with an AgNP / TPU and AgNP/WPU; Figure S13: TPU-based coatings deposited on polyethylene substrates.
