# Supplementary material for: Polyurethane-Based Coatings with Promising Antibacterial Properties
Source: Materials (Basel). 2020 Sep 25;13(19):4296. doi: 10.3390/ma13194296 (PMC7579457; doi:10.3390/ma13194296)
Supplement: Supplementary file 1 [file materials-13-04296-s001.pdf]

materials

Supplementary Material

Polyurethane-Based Coatings with Promising Antibacterial Properties

Maurizio Villani [1,*], Federico Bertoglio [2,3], Elisa Restivo [2], Giovanna Bruni [4], Stefano Iervese [2], Carla Renata Arciola [5,6], Francesco Carulli [1,7], Salvatore Iannace [1], Fabio Bertini [1] and Livia Visai [2,8,*]

1 Istituto di Scienze e Tecnologie Chimiche "Giulio Natta"—CNR, Via A. Corti 12, 20133 Milano, Italy; francesco.carulli@unimib.it (F.C.); salvatore.iannace@cnr.it (S.I.); fabio.bertini@scitec.cnr.it (F.B.)
2 Department of Molecular Medicine (DMM), Center for Health Technologies (CHT), UdR INSTM, University of Pavia, Viale Taramelli 3/B, 27100 Pavia, Italy; federico.bertoglio01@ateneopv.it (F.B.); elisa.restivo01@ateneopv.it (E.R.); stefano.iervese01@ateneopv.it (S.I.)
3 Technische Universität Braunschweig, Institute for Biochemistry, Biotechnology and Bioinformatics, Department of Biotechnology, 38106 Braunschweig, Germany
4 Center for Colloid and Surfaces Science (C.S.G.I.), Department of Chemistry, Physical Chemistry Section, University of Pavia, viale Taramelli 16, 27100 Pavia, Italy; giovanna.bruni@unipv.it
5 Department of Experimental, Diagnostic and Specialty Medicine (DIMES), University of Bologna, Via S. Giacomo, 14, 40126 Bologna, Italy; carlarenata.arciola@unibo.it
6 Laboratorio di Patologia delle Infezioni Associate all'Impianto, IRCCS Istituto Ortopedico Rizzoli, Via di Barbiano 1/10, 40136 Bologna, Italy
7 Dipartimento di Scienza dei Materiali, Università degli studi di Milano – Bicocca, Via Cozzi 55, 20125 Milano, Italy
8 Department of Occupational Medicine, Toxicology and Environmental Risks, Istituti Clinici Scientifici Maugeri S.p.A Società Benefit, IRCCS, Via S. Boezio, 28, 27100 Pavia, Italy
* Correspondence: maurizio.villani@scitec.cnr.it or mvillani078@gmail.com (M.V.); livia.visai@unipv.it (L.V.)

Received: 25 July 2020; Accepted: 23 September 2020; Published: date

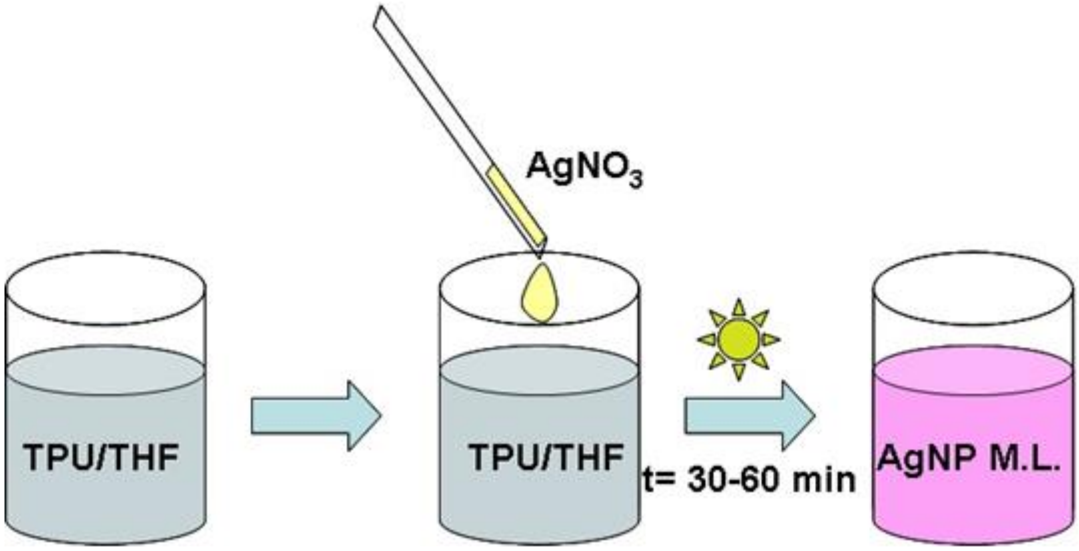

Figure S1. Schematic representation of the general procedure used for the preparation of M.L.

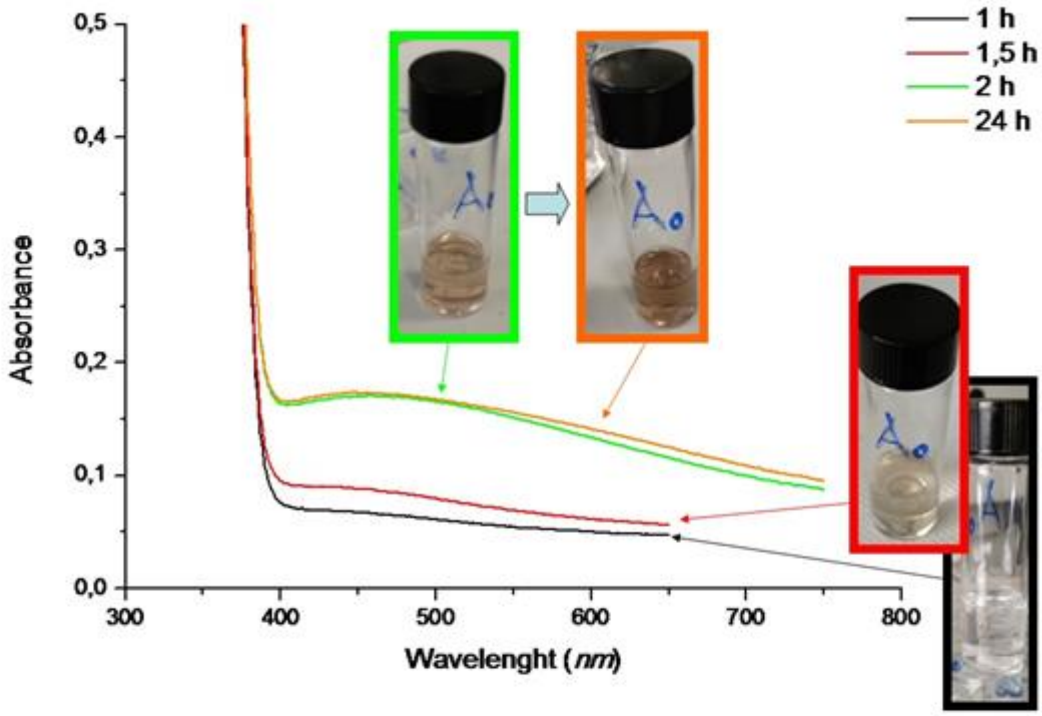

Figure S2. UV Absorption spectra as a function of exposure time to sun light for a M.L.1 sample.

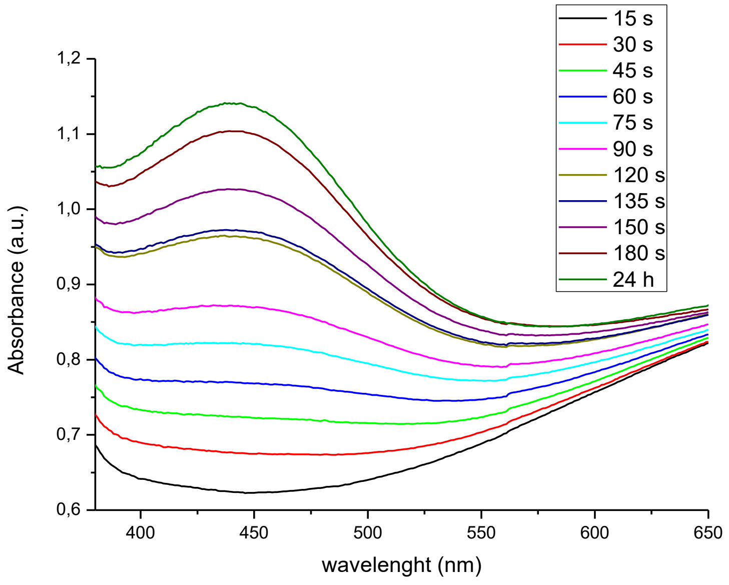

Figure S3. UV Absorption spectra as a function of exposure time to ultraviolet light for a samples at 50mM Ag precursor concentration.

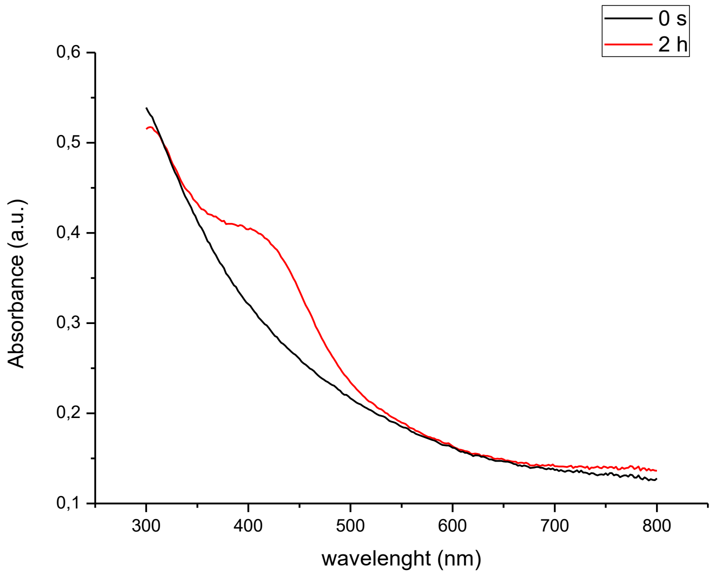

Figure S4. UV Absorption spectra as a function of exposure time to sun light for a film of M.L.5 prepared by spin coating deposition.

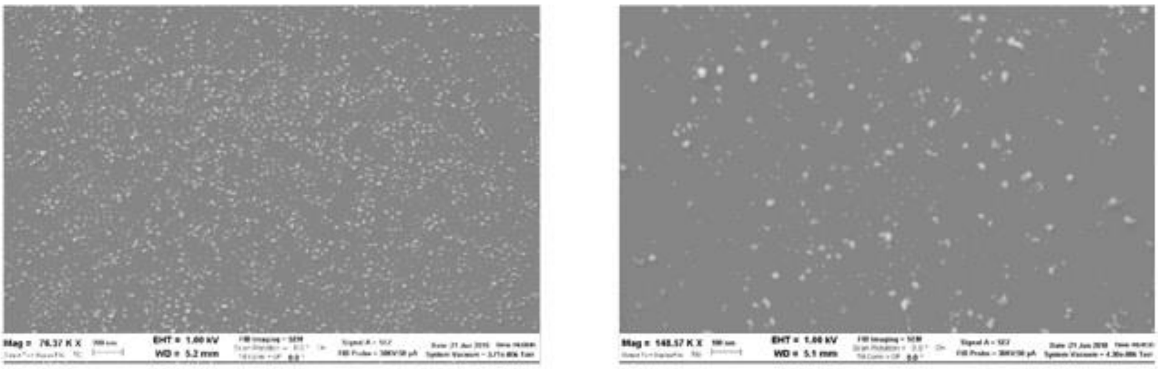

Figure S5. Surface morphology images of a homogeneous film obtained from M.L.1 and deposited by spin-coating at 76 kX (**a**) and at 148 kX (**b**) after plasma treatment.

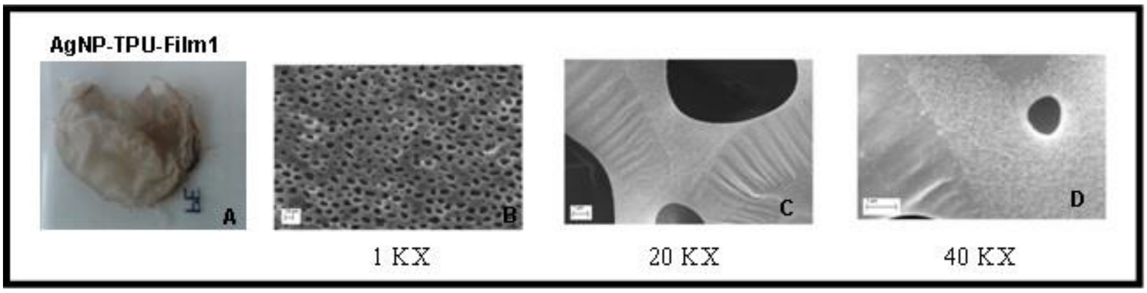

Figure S6. Photographic image of AgNP-TPU-Film1 (**A**) and surface morphology images of AgNP-TPU-Film1 at 1 kX (**B**), 20 kX (**C**) and 40 kX (**D**).

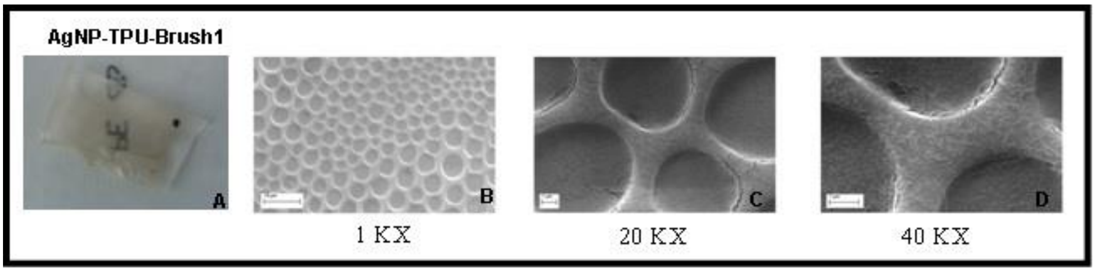

Figure S7. Photographic image of AgNP-TPU-Brush1 (**A**) and surface morphology images of AgNP-TPU-Brush1 at 1 kX (**B**), 20 kX (**C**) and 40 kX (**D**).

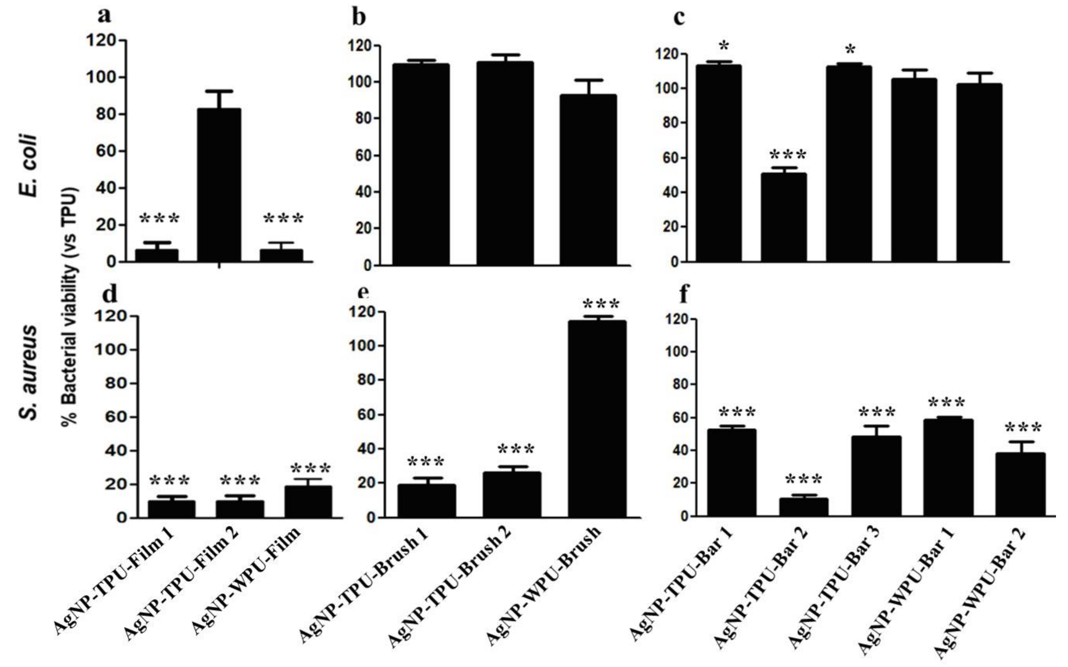

Figure S8. Planktonic bacterial viability through direct contact with materials. Comparison of *E. coli* ATCC 25922 (a-c) and *S. aureus* ATCC 25923 (d-f) viability with TPU set 100% after 6 h of incubation. Bars indicate mean values ± SD of the mean of results from two experiments. t-student test, statistical significance values were $P < 0.05$ (*) and $p < 0.001$ (***).

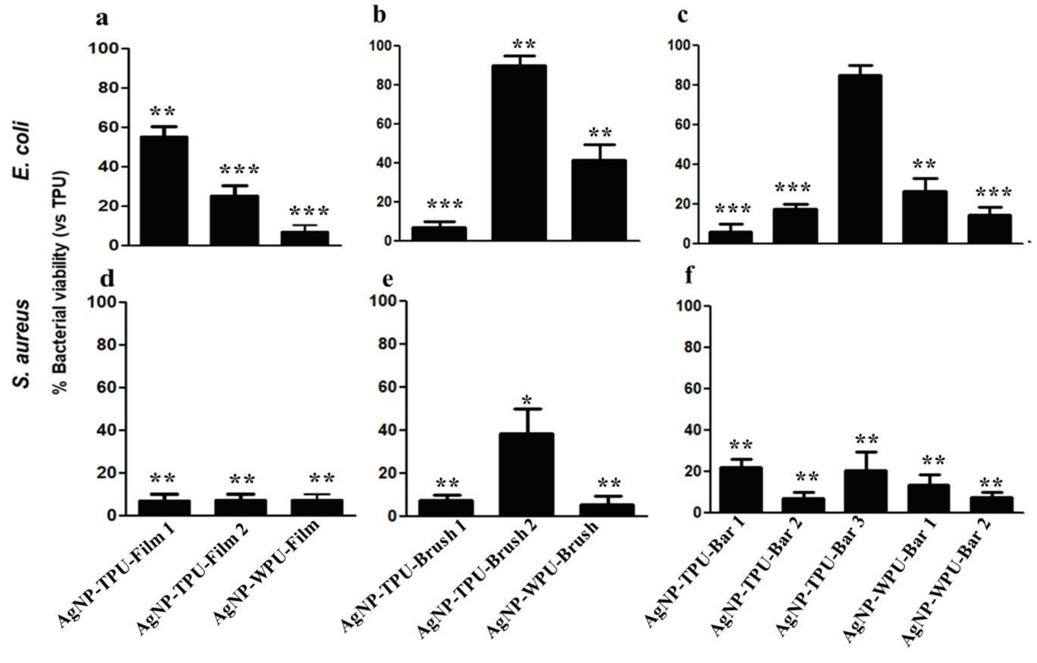

Figure S9. Bacterial adhesion on films through direct contact with materials. Comparison of *E. coli* ATCC 25922 (a-c) and *S. aureus* ATCC 25923 (d-f) viability with TPU set 100% after 6 h of incubation. Bars indicate mean values ± SD of the mean of results from two experiments. t-student test, statistical significance values were $P < 0.05$ (*), $p < 0.01$ (**) and $p < 0.001$ (***).

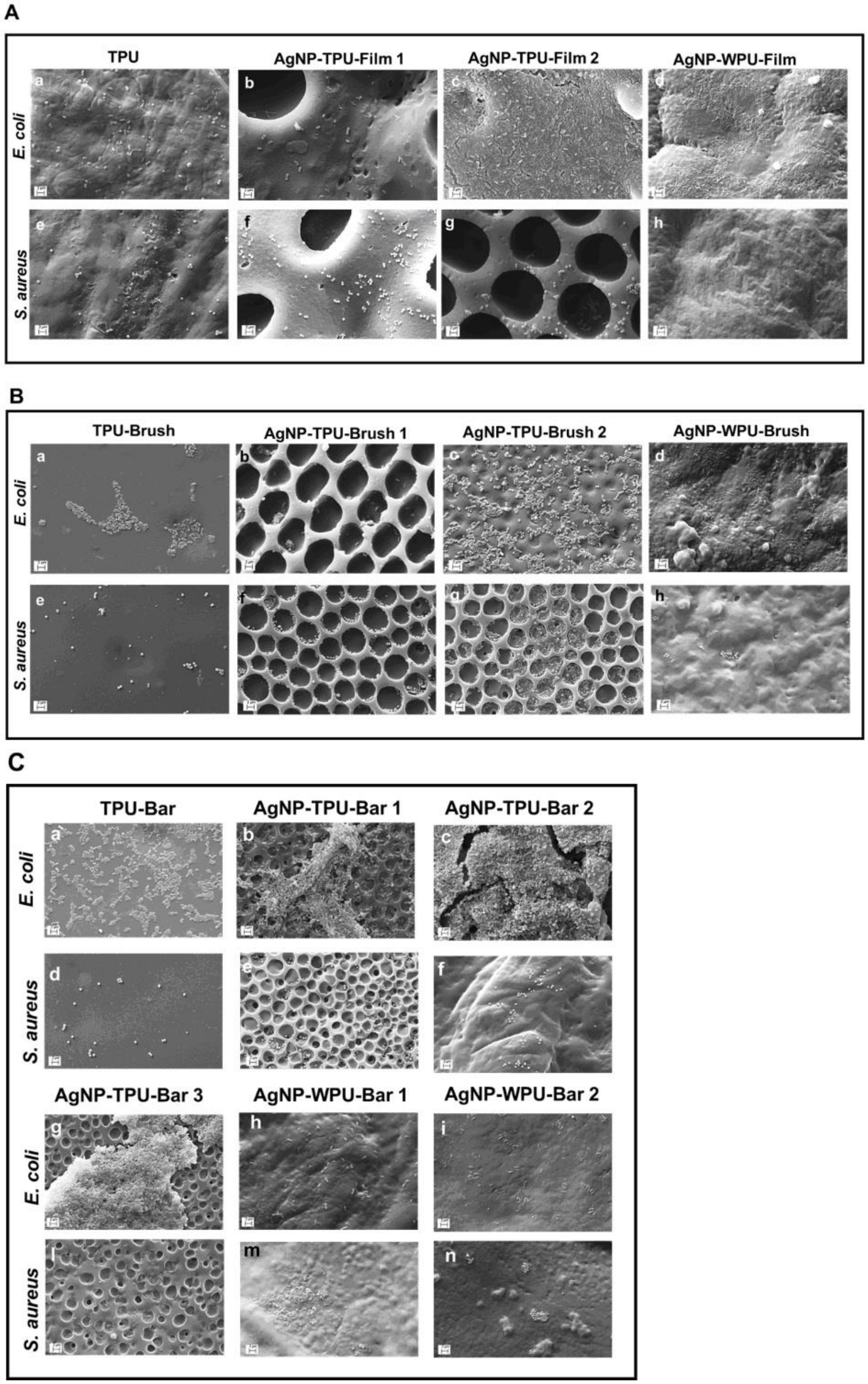

Figure S10. SEM images of bacteria adherent on TPU and WPU polymeric films at 3 kX (scale bar 10 µm). Panel A: *E. coli* (**a**–**d**), S. aureus (**e**–**h**) on TPU and casted films; panel B: *E. coli* (**a**–**d**), *S. aureus* (**e**–**h**) on brush films; panel C: *E. coli* (**a**–**c**; **g**–**i**), *S. aureus* (**d**–**f**; **l**–**n**) on bar coater films.

Table S1. pH values of the different reported samples after 24 h at 37°C measured in physiologic solution and Luria Bertani broth.

	pH	
	**Physiologic solution**	**Luria Bertani broth**
TPU	6	7
AgNP-TPU-Film 1	6	7
AgNP-TPU-Film 2	6	7
AgNP-WPU-Film	6	7
PU-Brush	6	7
AgNP-TPU-Brush 1	6	7
AgNP-TPU-Brush 2	6	8
AgNP-WPU-Brush	6	7
PU-Bar	6	7
AgNP-TPU-Bar 1	6	7
AgNP-TPU-Bar 2	6	7
AgNP-TPU-Bar 3	6	7
AgNP-WPU-Bar 1	6	7
AgNP-WPU-Bar 2	6	7

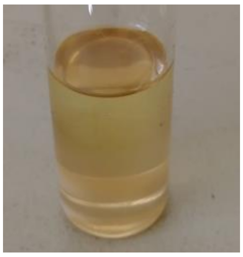

Figure S11. Photographic image of AgNP dispersed in a CHIT-solution after sun light exposition.

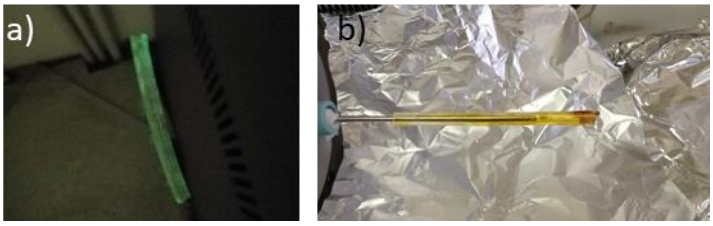

Figure S12. (**a**) prototype biomedical probe in polyurethane coated with an AgNP / TPU. To make the AgNP / TPU coating (tendentially pink / transparent) visible, a low amount of phosphorescent strontium aluminate was added. (**b**) prototype biomedical probe in polyurethane coated with an AgNP / WPU.

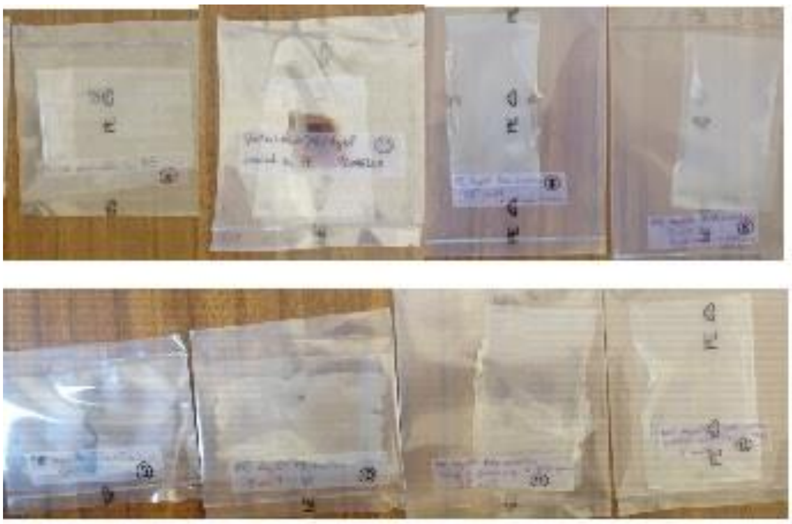

Figure S13. TPU-based coatings deposited on polyethylene substrates.

© 2020 by the authors. Licensee MDPI, Basel, Switzerland. This article is an open access article distributed under the terms and conditions of the Creative Commons Attribution (CC BY) license (http://creativecommons.org/licenses/by/4.0/).
